# Protocol for isolating stromal cells from lymphoid tissue for performing scRNA-seq

**DOI:** 10.1016/j.xpro.2026.104501

**Published:** 2026-04-15

**Authors:** Spyridon Makris, Daniel Shewring, Agnesska C. Benjamin, Sophie E. Acton

**Affiliations:** 1 Stromal Immunology Group, Laboratory for Molecular Cell Biology, University College London, Gower Street, WC1E 6BT London, UK

**Keywords:** Cell isolation, Single Cell, Cell separation/fractionation, Immunology

## Abstract

Stromal cells constitute less than 2% of cells in lymph nodes and are technically difficult to isolate and analyze. Here, we present a protocol to purify fibroblasts from lymphoid tissues in preparation for single-cell RNA sequencing (scRNA-seq) analysis. We describe steps for lymph node dissection and digestion, cell staining, and magnetic separation and also detailed procedures for checking purity and viability. This protocol can be applied to other tissues, including tumors.

For complete details on the use and execution of this protocol, please refer to Makris et al.^1^

## Highlights

• Procedures for dissecting and handling murine lymph node tissue
• Procedures for digesting lymphoid tissue to maximize viable stromal cells
• Procedures for sorting lymphoid stromal and immune cells for transcriptional analysis

## Abstract (editor highlights)

Publisher’s note: Undertaking any experimental protocol requires adherence to local institutional guidelines for laboratory safety and ethics.

## Graphical abstract

**Figure:**
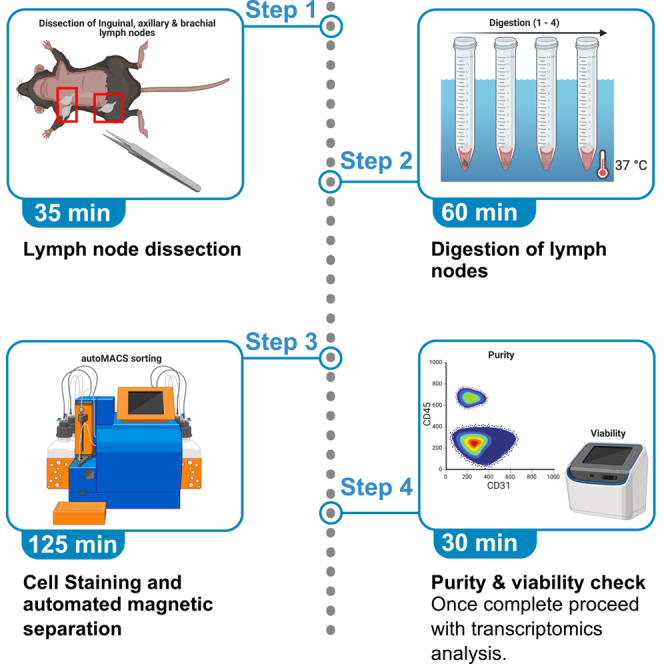

## Before you begin

The protocol below describes the specific steps for collecting lymph nodes, isolating cells and maintaining cell viability. This protocol was optimized to perform scRNA-seq analysis and flow cytometry analysis^1^ but can also be used for studying primary fibroblasts *in vitro*. It provides a detailed description of how lymph nodes should be dissected and handled prior to tissue digestion. The digestion method described ensures the successful isolation of all lymphoid cells, stromal and immune cell populations. The usage of magnetic separation (automated or magnetic columns can be used) is used to purify fibroblasts to achieve a sufficient yield and viability for performing scRNA-seq analysis. The protocol described is focused on skin draining lymph nodes however it can be applied to other tissues, including solid tumors,^2^ where stromal cell function is of increasing interest.

### Innovation

Stromal cells have commonly been described for their structural and mechanical properties across tissues.^3^^,^^4^^,^^5^^,^^6^^,^^7^ More recent research places stromal cells as important immunoregulatory cells in health and disease.^1^^,^^2^^,^^3^^,^^7^^,^^8^^,^^9^ These cells are often overlooked due to their scarcity combined with technical challenges when isolating them. This protocol expands on previously described lymph node dissection and digestion methods^10^^,^^11^^,^^12^ and focuses on the technical challenges encountered during tissue dissection, digestion and isolation of stromal cells. The protocol expands on previous publications by providing precise methods of dissecting lymph nodes and handling tissue during the digestions.^12^ The methods described also ensure higher recovery of stromal cells compared to other published protocols and an improved sequencing depth for scRNA-sequencing.^11^^,^^13^^,^^14^

Specifically, we provide a detailed description of the technical skills required for dissecting and handling murine lymph nodes. Our findings indicate that ensuring a sufficient yield of viable cells requires a combination of delicate sample handling and speed. Using autoMACS® our approach achieves a sufficient yield, and purity, of scarce stromal cells whilst maintaining cell viability ensuring successful analysis via in depth scRNA-seq analysis.

### Institutional permissions

Experiments were performed in accordance with national and institutional guidelines for animal care and approved for the Laboratory for Molecular and Cell Biology by the institutional animal ethics committee review board, European research council, and the UK Home Office. Mice aged 8–10 weeks were used for these experiments.

### Preparation of digestion buffer enzymes

**Timing: 1 h**

1. Prepare Dispase II aliquots (100 mg/mL).
  a. Dissolve 5 g of Dispase II into 50 mL of distilled phosphate-buffered saline (PBS) and gradually and gently mix using a vortex on very low speed.
  b. Mix well by inverting a few times.
  c. Once dissolved, prepare 0.5 mL aliquots in 1.5 mL centrifuge tubes and store at −80°C for up to 12 months.

**CRITICAL:** Dispase II may clump when reconstituted therefore attempt to dissolve in smaller volumes and gradually transfer into a 50 mL Falcon tube.

2. Preparation of DNase I (10 mg/mL).
  a. Dissolve 100 mg of DNase I into 10 mL of distilled water.
  b. Gradually mix using 2.5 mL of distilled water and transfer into a 15 mL Falcon tube.
  c. Prepare 0.5 mL aliquots in 1.5 mL centrifuge tubes and store at −80°C for up to 12 months.

***Note:*** Collagenase is always used fresh when preparing the Digestion Buffer immediately prior to starting tissue digestion. This approach minimizes variability between experiments.

### Preparation of FACS and MACS buffers

**Timing: 1 h**

3. Preparation of FACS buffer.
  a. Add 1% Bovine Serum Albumin (BSA) to PBS in glass bottle and mix using magnetic stirrer.
  b. Once BSA dissolves add 5 mM ethylenediaminetetraacetic acid (EDTA) and gently mix.
  c. Store Buffer at 4°C for up to 7 days.
4. Preparation of MACS buffer.
  a. Add 0.5% of Fetal Bovine Serum (FBS) into PBS in glass bottle and mix.
  b. Add 2 mM EDTA and resuspend.
  c. Store MACS buffer at 4°C for up to 7 days.

### Preparation of processing tubes

**Timing: 0.5 h**

**Figure 1:**
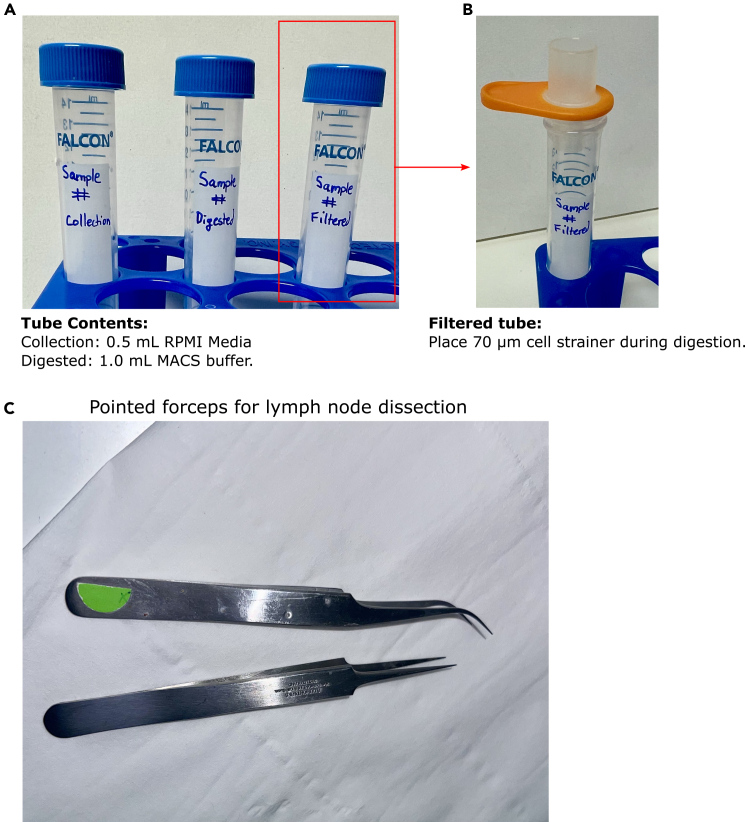
Preparation of tissue processing tubes and instrumentation (A) Labelling of “Collection,” “Digested” and “Filtered” tubes for each sample. (B) Filtering tube with 70 μm cell strainer. (C) Example of pointed forceps used for dissection of lymph nodes.
  c. Add 0.5 mL Roswell Park Memorial Institute (RPMI) media into “Collection” tube and store at 4°C for up to 72 h before experiment.
  d. Add 1 mL MACS buffer to ‘Digested’ tube and store at 4°C for up to 72 h before experiment.
  e. Prior to commencing digestion place 0.70 μm filter on “Filtered” tube (Figure 1B).

***Note:*** Preparation of tubes prior to experiment is essential for optimizing speed of tissue processing.

5. Preparation of processing tubes.
  a. Prepare 3 x 15 mL Falcon tubes for each sample collected.
  b. Label tubes with sample number and “Collection”, “Digested” and “Filtered” (Figure 1A).

**CRITICAL:** Do not use FBS or BSA in collection tube as it will affect the efficacy of digestion enzymes.

## Key resources table

**Table:** 

REAGENT or RESOURCE	SOURCE	IDENTIFIER
**Antibodies**		

Rat anti-mouse CD16/CD32 (Fc-Block) (Dilutions in 1:200)	BD Biosciences	Cat# 553141, RRID:AB_394656
CD45 antibody, anti-mouse, Biotin (Dilutions in 1:50)	Miltenyi Biotec	Cat# 130-124-209, RRID:AB_2819580
CD31 antibody, anti-mouse, Biotin (Dilutions in 1:50)	Miltenyi Biotec	Cat# 130-119-562, RRID:AB_2751728
Anti-Biotin MircoBeads (Dilutions in 1:50)	Miltenyi Biotec	Cat# 130-090-485, RRID:AB_244365
CD45 AF700 (dilution 1:100)	BioLegend	Cat# 103127, RRID:AB_493714
Aqua™ Fixable Viability Kit 500 tests	BioLegend	Cat# 423102, RRID: N/A
CD31 PerCP-Cy5.5 (dilutions in 1:50)	BD Biosciences	Cat# 562861, RRID:AB_2737847
CD31 APC/Fire™ 750 (dilution in 1:50)	BioLegend	Cat#102434, RRID:AB_2629683
Podoplanin monoclonoal antibody – host: Hamster – clone: 811 (Dilutions in 1:500)	Novus	Cat#DM3501; RRID:AM2161936
DAPI (Dilutions in 1:1000)	Merck	Cat# 28717-90-30
Hoechst 33342 Solution (20 mM) (Dilutions in 1:2000)	Fisher Scientific	Cat#10150888
Goat anti-Hamster IgG (H+L) Cross-Absorbed Secondary Antibody, AF488 (Dilutions in 1:500)	ThermoFisher Scientific	Cat# A-21110; RRID:AB_141509

**Chemicals, peptides, and recombinant proteins**		

Collagenase D	Sigma-Aldrich	Cat# 11088866001
Dispase II	Thermo Scientific	Cat# 17105041
DNase I, Grade II, from bovine pancreas	Roche	Cat# 10104159001
EDTA (0.5 M), pH 8.0, RNase-free	ThermoFisher Scientific	Cat# AM9260G
RPMI Medium 1640 (1X)	gibco	Cat# 11875-093
Fetal Bovine Serum Heat Inactivated, 500 mL	Sigma-Aldrich	Cat# F9665-500ML
Bovine Serum Albumin Lyophilized Powder, 50 G	Sigma-Aldrich	Cat# A2153-50G

**Software and algorithms**		

Fiji, ImageJ	Schindelin et al.^15^	https://doi.org/10.1038/nmeth.2019
Prism10 Software	Graphpad	https://www.graphpad.com/scientific-software/prism/
FlowJo Software	BD Biosciences	https://www.flowjo.com/solutions/flowjo

**Other**		

15 mL High-Clarity Polypropylene Conical Tube	Falcon	Cat# 352096
Reversible Strainer, 70 μm mesh	STEMCELL Technologies	Cat# 27216
BD LSR II 3 Laser, Blue/Red/Violet	BD Biosciences	Cat# 33300456
LUNA-FX7™ Automated Cell Counter	Logos Biosystems	Cat# L70001
LUNA™ 8-channel slides	Logos Biosystems	Cat# L72001
LUNA™ 2-channel slides	Logos Biosystems	Cat# L12001
Acridine Orange/Propidium Iodine stain	Logos Biosystems	Cat# F23001
Trypan Blue Stain, 0.4%, Sterile-filtered	Logos Biosystems	Cat# T13011
Leica TCS SP8 STED 3 X confocal microscope	Leica Microsystems	Cat# N/A
autoMACS® Pro Separator	Miltenyi Biotec	Cat# 130-092-545
Isotemp™ GPD 05 Water Bath	Fisher Scientific	Cat#15375877
Spring Forceps curved 125mm	VWR	Cat# 232-0037
S Murray and Co™ Stainless Steel Watchmakers Forceps	Fisher Scientific	Cat# 12311379
Centrifuge 590 Ri	Eppendorf	Cat# 17111852
Precision Wipes – White/Small	Kimtech Science	Cat# 07552000
96 well clear round bottom polypropylene plates	Corning	Cat# 3879
Fisherbrand™ Microplate Shaker	Fisher Scientific	Cat# 15504070
Cytospin 4 Cytocentrifuge	Epredia	Cat# A78300002
Single Cytoslides	Epredia	Cat# B1000731
Single Cytofunnel™, White	Epredia	Cat# 5991040
Cytoclip™ Stainless Steel Slide Clips	Epredia	Cat# 59910052
Cover glasses, Manzel Gläser	Epredia	Cat# 12362128
21 g Green 1.5″ Microlance Needles	BD	Cat# 10472204
Ethanol absolute EMPLURA®	Merck	Cat# 8187602500
autoMACS® Running buffer – MACS Separation Buffer	Miltenyi Biotec	Cat# 130-091-221
autoMACS® Pro Washing Solution	Miltenyi Biotec	Cat# 120-092-987
autoMACS® Columns (5×2)	Miltenyi Biotec	Cat# 130-021-101

## Materials and equipment

### Buffer recipes

**Table:** Digestion Buffer

Reagent	Final concentration	Amount
Dispase II aliquots (100 mg/mL)	10 mg/ml	0.5 mL
DNase I aliquots (100 mg/mL)	10 mg/mL	0.5 mL
Collagenase D	0.25 mg/mL	12.5 mg
PBS	1x	48 mL
Total	N/A	50 mL

Prepare just before digestion begins and strictly maintain on ice. Approximately 8 mL is required for each sample.

**CRITICAL:** Trypsin can be present within batches of collagenase enzymes and can affect cell detection and cause cell damage. Seek commercially supplied enzymes with defined consistent composition to maximize stromal cell viability.

**Table:** FACS Buffer

Reagent	Final concentration	Amount
BSA	1%	5 g
EDTA (0.5 M)	5 mM	5 mL
PBS	1x	499.5 mL
Total	N/A	500 mL

Store at 4°C for up to 7 days.

**Table:** MACS Buffer

Reagent	Final concentration	Amount
FBS	0.5%	0.25 mL
EDTA (0.5 M)	2 mM	0.20 mL
PBS	1x	49.55 mL
Total	N/A	50 mL

Store at 4°C for up to 3 days. Approximately 50 mL of MACS buffer is required for each experimental group.

**RPMI Media 2% FBS:** Add 1 mL of FBS into 49 mL of RPMI to achieve 2% FBS concentration.

## Step-by-step method details

### Part 1: Collection of lymph nodes

**Timing: 35 min**

In this step, lymph nodes are collected from mice and prepared for digestion. Timings are calculated from starting of mouse culling until collection of the inguinal, axillary and brachial lymph nodes from each side of 6 naïve C57BL/6 mice. Inguinal, axillary and brachial lymph nodes were collected and combined for scRNA-seq analysis presented in Makris et al.^1^ To minimize tissue damage and ensure stromal cell viability use pointed forceps for dissecting lymph nodes (Figure 1C). Preparation of mice in step 1, and removal of fat tissue from lymph nodes in step 2 will ensure a sufficient yield of fibroblasts.

**Figure 2:**
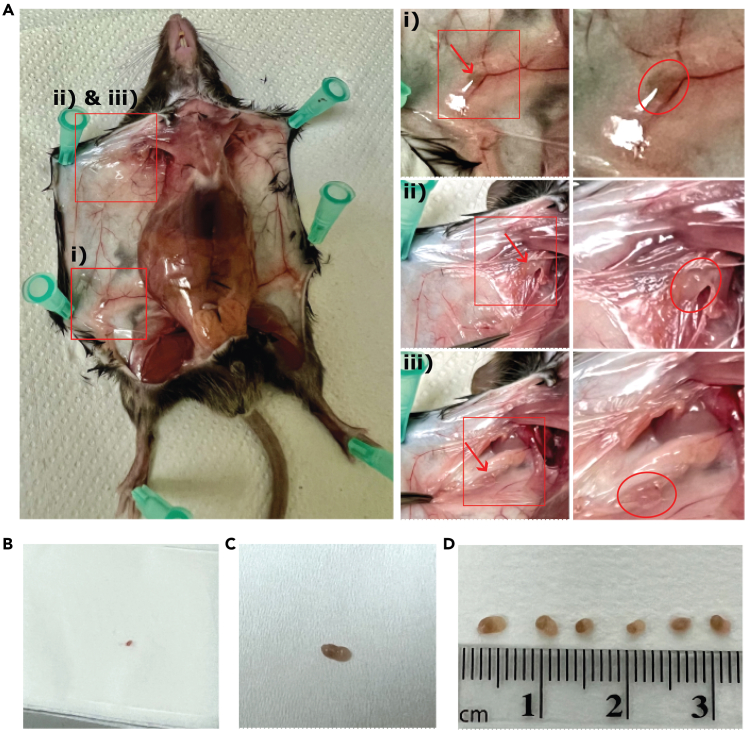
Dissection of inguinal, axillary and brachial lymph nodes (A) Pinning of mouse on dissection board and identification of (i) inguinal, (ii) axillary and (iii) brachial lymph nodes. Red arrow and outline show the position of each lymph node. (B) Collected lymph node on Precision Wipes. (C) Inguinal lymph node after removal of fat and connective tissue. (D) Representative size of inguinal lymph nodes collected from naïve mice.
  c. Spray each mouse with water to dampen fur.
  d. Carefully cut the skin along the center of the lower abdomen towards the neck while avoiding perforating the abdominal cavity.
  e. Expose lymph nodes by pinning skin as shown in Figure 2A.

1. Preparation of mouse for collecting lymph nodes.
  a. Euthanize mice using CO_2_ overdose.
  b. Pin mouse to a dissection board aligning the limbs and making sure not to stretch excessively (Figure 2A).

***Note:***Figure 2A shows location of the lymph nodes on right flank of mouse. The locations are identical on the left flank.

2. Collection of inguinal lymph nodes.
  a. Using fine pointed forceps (Figure 1C) separate and lift the inguinal lymph nodes from fat pads (Figure 2A(i)).
    **CRITICAL:** It is important to handle the lymph node very gently, without squeezing tissue in forceps, as fibroblast yield may reduce significantly.
  b. Gently turn lymph nodes over on Precision Wipes (Figure 2B) ensuring not to tear or damage the nodes and remove any excess fat or connective tissue (Figure 2C).
    **CRITICAL:** Remove all excess fat or connective tissue as this will reduce yield during digestion.
  c. Place into respective “Collection” tube containing 0.5 mL of RPMI.
    ***Note:*** For research comparing treatment groups, the collected lymph nodes can also be weighed before transferring to the “Collection” tube. Lymph node weights and sizes can vary between mice and treatment groups (e.g. naïve versus reactive lymph nodes) (Figure 2D). Stromal-to-Immune cell ratios will vary in reactive lymph nodes.
3. Collection of axillary and brachial lymph nodes.
  a. Remove axillary lymph nodes (Figure 2A(ii)) as described in steps 1a–c, ensuring not to puncture proximal blood vessels. Presence of blood within lymph nodes will decrease reduce cellular yield.
  b. Move thin tissue layer to expose brachial lymph node in the underarm area (Figure 2A(iii)) and collect as described in steps 2a–c.

**CRITICAL:** Maintain all samples on ice until digestion step.

### Part 2: Digestion of lymph nodes and extraction of single-cell suspension

**Timing: 75 min**

**Figure 3:**
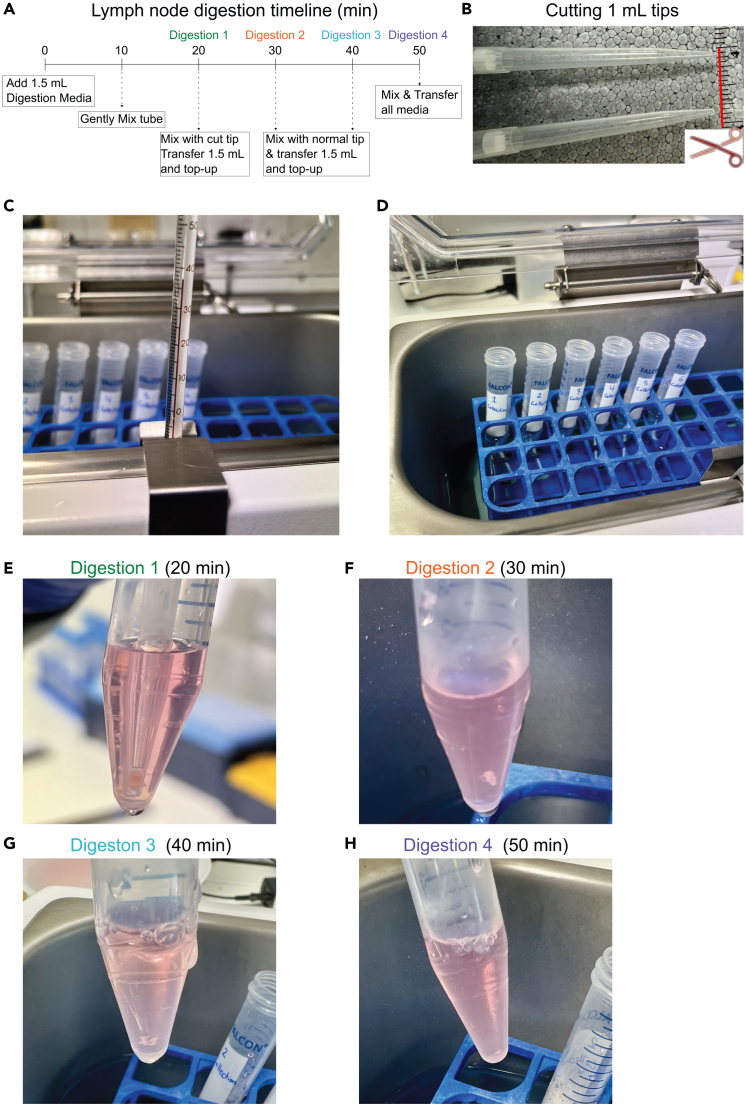
Lymph node digestion (A) Diagram showing timeline of lymph node digestion. (B) Cutting of 1 mL pipette tip in accordance with the size of collected lymph node. (C) External thermometer indicating stabilized water temperature at 37°C. (D) Samples placed in water bath and digestion buffer added. (E) Digestion of lymph node after 20 min where lymph node is pipetted in cut 1 mL tip. (F) Digestion 2 collected at 30 min. (G) Digestion 3 collected at 40 min. (H) Digestion 4 at 50 min where no tissue is visible.

In this step, collected lymph nodes will be digested, and isolated cells will be filtered. The protocol describes the optimal methods for handling the tissue and timings for digestion steps which ensure viability of stromal and immune cells. Lymph nodes are gently mixed at regular intervals and digestion mix is replaced until lymph node is fully dissociated (Figure 3A).

***Note:*** Immediately prior to starting digestion, or during initial 20 min incubation (step 5), cut 1mL pipette tips to widen the tip opening according to lymph node size (Figure 3B). This will ensure gentle passage of lymph node through the tip and increase stromal cell viability.

4. Set water bath to exactly 37°C and use external thermometer to ensure temperature is appropriate (Figure 3C).

**CRITICAL:** Ensure the temperature is precisely at 37°C. Digestion enzyme activity is greatly affected by temperature. Higher temperature causes increased digestion speed and lower temperature will decrease digestion speed. Non-optimal temperature will significantly affect stromal cell viability (see troubleshooting section – Figure 7).

5. Prepare the Digestion Media and add 1.5 mL into each of the collection tubes and incubate in water bath for 20 min (Figure 3D).
  a. At 10 min gently tap and shake each tube and place back into water bath.

**CRITICAL:** Prepare Digestion Media just before starting the digestion protocol and strictly maintain on ice.

6. After the 20 min incubation, pipette and gently move the lymph node through the cut 1 mL pipette tip 5 times (Figure 3D).
7. Once lymph nodes have settled back to the bottom of tube (Figure 3E), remove 1.5 mL of the digestion media, containing the first cell fraction and transfer to “Digested” tubes and maintain on ice.

***Note:*** Depending on the experimental objectives, “Digested” tubes can either contain FACS buffer (for flow cytometry) or MACS buffer (for MACS sorting).

8. Add 1.5 mL of fresh digestion media to the samples and incubate for a further 10 min in the water bath at 37°C.
9. Now switch to uncut 1 mL pipette tip and repeat steps 5–7 (Figures 3G and 3H).

***Note:*** Lymph nodes digestion time is approximately 50 min.

10. Once there is no visible tissue remaining transfer all remaining digested cell suspension to the “Digested” tube and centrifuge at 350 *g* for 10 min at 4°C.

**Figure 4:**
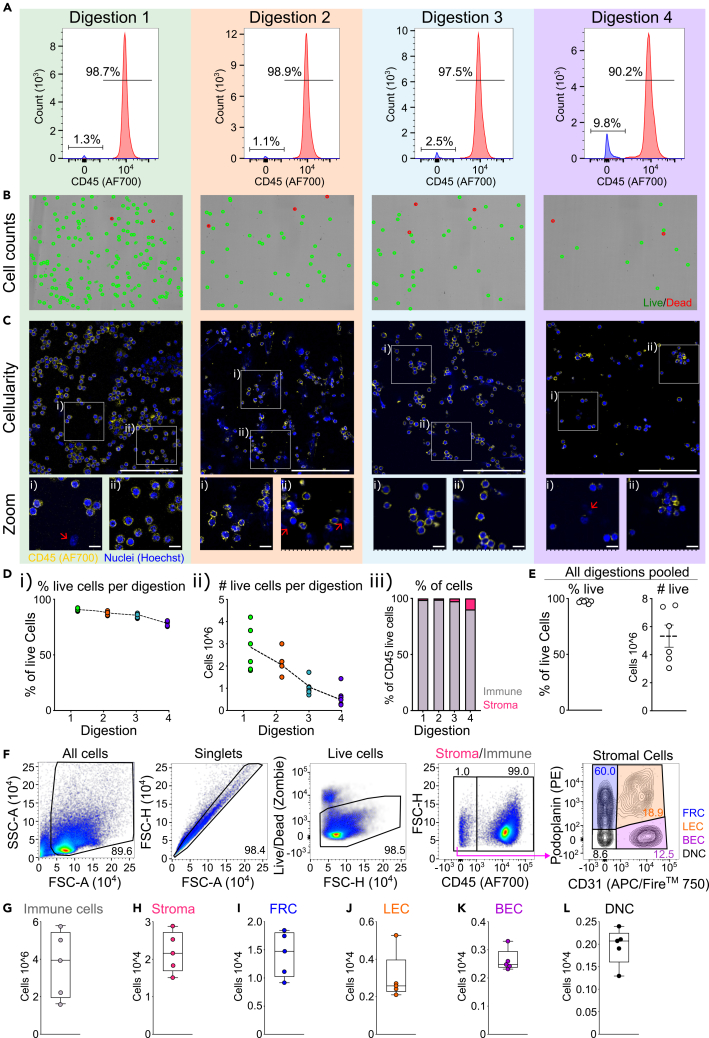
Composition of cells recovered after each digestion step Collected cells were analyzed after each digestion step to determine cell phenotypes, numbers and viability. When performing transcriptomics analysis all digestion fractions will be combined. (A) Percentages of immune cells (CD45^+^ cells) and stromal cells (CD45^-^ cells) extracted from each digestion step analyzed via flow cytometry. (B) Representative viability images of each digestion step acquired on automated cell counter (LUNA 7-FX™) where red indicates dead cells and green live cells. (C) Immunofluorescence images of cells stained after each digestion step for CD45 (yellow) and Nuclei (blue). Representative Cytospin slides. Red arrows showing examples of CD45^-^ cells. Scale bars indicate 100 μm for main images and 10 μm for zoom. (D) After each digestion step the (i) percentage of live cells, (ii) number of live cells after each digestion measured by automated cell counting and (iii) percentage of immune (gray) and stromal cells (red) after each digestion fraction. (E) Combined cells from all digestions showing percentage and number of cells from each lymph node. (F) Representative gating strategy for lymph node cells showing stromal cell percentages. (G–L) Number of (G) immune cells, (H) stromal cells, (I) fibroblastic reticular cells (FRC), (J) lymphatic endothelial cells (LEC), (K) blood endothelial cells (BEC) and (L) double negative cells (DNC).

***Note:*** Centrifuging at low speed and longer time minimizes cell loss and maximizes viability.

**CRITICAL:** It is important to fully digest the tissue to acquire enough stromal cells (Figure 4).

11. Gently resuspend cell pellet in 3 mL of MACS buffer and filter through 70 μm cell strainer into the “Filtered” tube.
  a. If performing flow cytometry instead of automated cell sorting use FACS buffer.

***Note:*** The main populations of stromal cells expected from each lymph node are fibroblastic reticular cells (FRC), lymphatic endothelial cells (LEC), blood endothelial cells (BEC) and double negative cells (DNC) (Figures 4F–4L).

12. Count cells using automated cell counter.

***Note:*** For Makris et al.^1^ the LUNA-FX7™ Automated Cell Counter was used and cells were diluted 1:10 in 0.2% trypan blue (Figure 4B).

13. Centrifuge samples at 350 *g* for 10 min at 4°C and resuspend at desired concentration (see part 3).

### Part 3: Purification of fibroblasts using automated magnetic separation

**Timing: 125 min**

In this step, isolated lymph node cells are sorted using the Miltenyi autoMACS® cell separator. To accomplish a sufficient yield of cells pool lymph nodes from mice or treatment groups. For negative selection of fibroblasts, cell suspension is stained with CD45-biotin and CD31-biotin conjugated antibodies to positively select immune and endothelial cells. This creates a “positive” fraction with immune and endothelial cells and a negative target-fraction containing fibroblasts.

**CRITICAL:** For all incubations steps in Part 3, samples must be maintained on ice and placed on a shaker on lowest speed setting. This will ensure cells do not clump and will maximize antibody binding.

***Note:*** Keeping cells on ice maintains a more consistent temperature than 4°C in the fridge.

14. Block cells using Rat anti-mouse CD16/CD32 (Fc-Block) (1:200) in MACS buffer for 15 min on ice. Use 95 μL of MACS buffer with 5 μL of Fc-block for every 10^7^ cells.
  a. During this incubation prepare primary antibodies used in step 16.
15. To wash the cells, add an additional 2 mL of MACS buffer per 10ˆ7 cells and centrifuge for 10 min at 350 *g* at 4°C.
  a. Repeat this step once for a total of 2 washes.
16. Stain with primary antibodies and keep on ice for 20 min.
  a. Resuspend 10^6^ cells/98 μL of MACS buffer containing CD45-biotin and CD31-biotin antibodies diluted at 1:50.

***Note:*** Antibodies used in this example are supplied at a concentration of 150 μg/mL and dilutions based on manufacturer’s recommendation which is 1:50.

**Figure 5:**
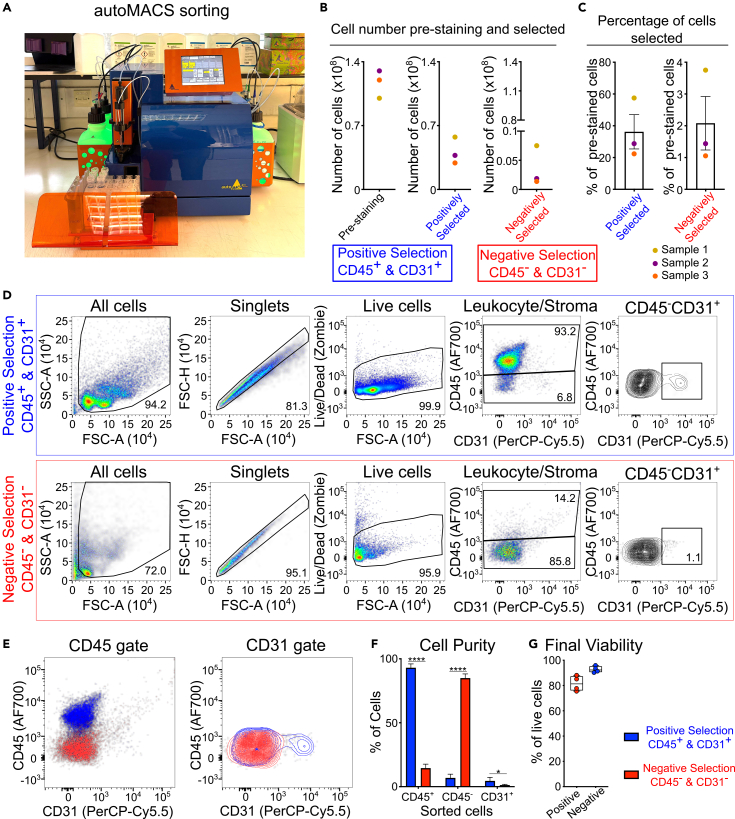
Cell selection using automated magnetic cell sorting (A) Cells were stained with CD45-biotin and CD31-biotin and sorted using autoMACS® Pro Separator. (B) Number of cells before staining for autoMACS® separation (step 11), and number of cells recovered from positive selection (CD45^+^ and CD31^+^ cells) and negative selection (CD45^-^ and CD31^-^ cells) in step 23. Each dot represents combined numbers from inguinal, axillary and brachial lymph nodes from 6 mice. Colors indicate biological replicates. (C) Percentages of cells after separation compared to the pre-staining cell count performed in step 11. (D) Purity check of separated cells using flow cytometry and staining for CD45 and CD31. (E) Overlay plots of positive selection (blue) and negative selection (red). (F) Percentage of CD45^+^, CD45^-^ and CD31^+^ cells recovered (∗∗∗∗ p value < 0.0001, ∗ p value < 0.05). (G) Final viability check of positive and negative selected cells acquired just before performing scRNA-sequencing analysis.

***Note:*** For sorting 15 mL Falcon tubes were used. Other tube sizes can be used during magnetic sorting following manufacturer’s recommendations.

17. Wash cells as in step 15.
18. Stain cells with anti-biotin beads and incubate for 15 min on ice.
  a. For every 10^7^ cells add 80 μL of MACS buffer and 20 μL of anti-biotin beads.
19. Wash cells as in step 15.
20. Resuspend cells at 0.5 mL of MACS buffer per 10^8^ cells.
  a. If cell number in any single sample is lower than 10^8^, then resuspend in a minimum of 0.55 mL for autoMACS sorting.
  b. Transfer a small aliquot (typically 50 μL) into a 1.5 mL centrifuge tube to quantify pre-sorting purity (see part 4).
21. Run autoMACS (Figure 5A) program following manufacturer’s instructions.
  a. For fibroblast purification the ‘POSSEL’ standard separation program can be used.
  b. A sorting run takes approximately 5 min per sample.
  c. Positive selection will include CD45^+^ and CD31^+^ cells while negative selection will contain fibroblasts (CD45^-^CD31^-^).

22. Centrifuge collected cells at 350 *g* for 10 min at 4°C.
  a. Top-up each sample with RPMI Media 2% FBS so that volume of each sample is approximately 10 mL before spinning.
23. Resuspend samples in RPMI Media 2% FBS.
  a. Resuspend samples at a concentration of 10^7^ cells per mL.
24. Count cells using an automated cell counter and record cell viability (Figure 5B).

***Note:*** The number of cells will be reduced during the staining and washing steps (Figure 5C). Prepare samples based on the number of cells required for further transcriptomics assays and use Figure 5C to estimate the cell yield.

***Note:*** Using fluorescently labelling viability stain such as Acridine Orange/Propidium Iodine stain (following manufacturer’s instructions) rather than trypan blue will be more accurate in this step especially for negatively selected cells.

25. Remove aliquot from each cell population and proceed to purification check.
  a. Transfer 100 μL from positively selected cells into a 1.5 mL centrifuge tube.
  b. Transfer up to 30–50 μL from negative selected cells into a 1.5 mL centrifuge tube.
26. Remaining cells should be stored on ice until quality control and purity check has been completed.

### Part 4: Purity check of sorted cells

**Timing: 30 min**

In this step, separated cells will be checked for purity. This will ensure the enrichment of fibroblasts which can then be used for transcriptomic analysis. The purity check only uses a small aliquot of the cells and a low number will be recorded at a low acquisition speed. Ensure to proceed with this step as promptly as possible to maintain viability of cells in step 26.

27. Place collected pre-sorted and post-sorted cells into U-bottom 96-well plate.
  a. Add FACS buffer and bring total volume to approximately 200 μL per well.
  b. Spin at 500 *g* for 2 min and then remove supernatant by gently flicking the plate.

**CRITICAL:** Very gently flick the plate in this step making sure not to disrupt cell pellets and do not blot.

***Note:*** Cell pellet in negative selection might not be visible.

28. Resuspend cells in 50 μl of staining solution and incubate on ice for 20 min on ice.
  a. CD45 antibody at 1:100 dilution in FACS buffer.
  b. CD31 antibody at 1:50 dilution in FACS buffer.
29. Wash cells by adding 150 μL of FACS buffer and spin as in step 27.
  a. Resuspend cells in 200 μL of FACS buffer.
30. On a flow cytometer check the purity of separated cells by gating CD45^+^ and CD45^−^ and CD45^−^CD31^+^ cells (Figures 5D–5F).
  a. Proceed with sequencing of sorted cells from step 26.

***Note:*** Also check the purity of CD31 staining in the positively selection. A low number of cells is expected.

31. Preparation of cells for transcriptomics analysis.
  a. Before sequencing centrifuge cells once at 350 *g* for 10 min at 4°C.
  b. Perform a cell count and viability check (Figure 5G).
  c. Resuspend cells at desired concentration based on the sequencing protocol used.

***Note:*** Cell number and viability thresholds may differ based on the transcriptional analysis method required. In Makris et al.^1^ samples were labelled on the 10X Chromium Platform.

## Expected outcomes

**Figure 6:**
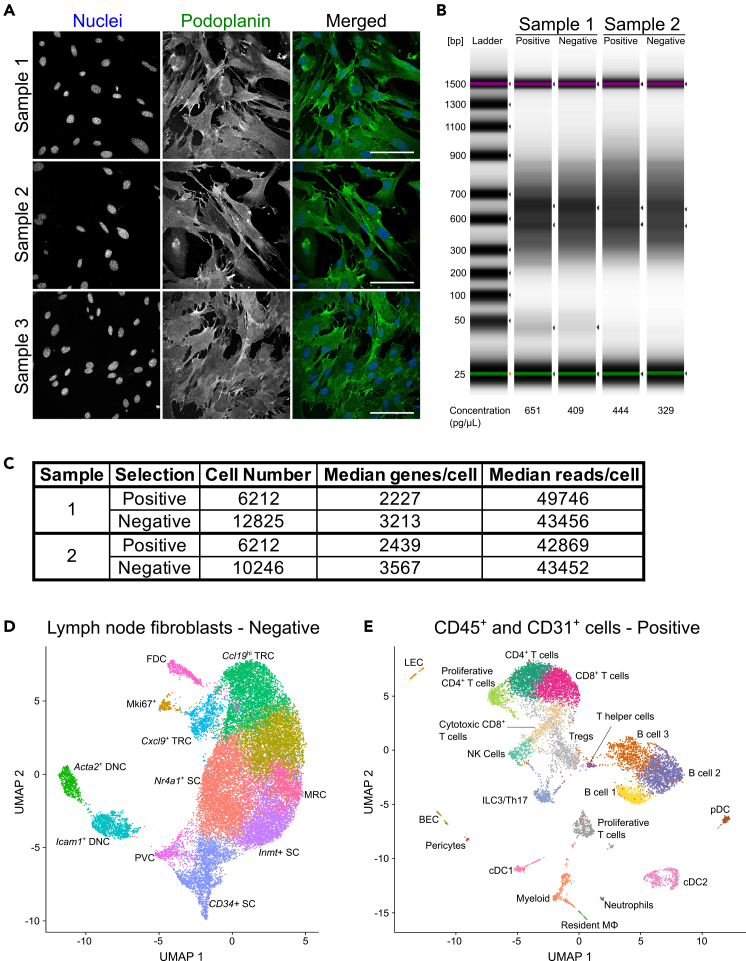
Outputs from digestion of lymph nodes and scRNA-seq analysis Cells extracted from lymph nodes can be used for flow cytometry analysis (Figure 4), primary cell culture or scRNA-sequencing. (A) Representative images of primary fibroblasts stained for nuclei (DAPI – blue) and podoplanin (green). Scale bar = 50 μm. (B and C) Outputs from scRNA-seq analysis using 10x Chromium platform showing (B) cDNA quality control, (C) number of cells captured, median genes/cell and median reads/cell. (D and E) Analysis of scRNA-sequencing data showing Unifold Manifold Approximation and Projection (UMAP) plots for negative and positive selections Data for C–E is from Makris et al., 2025 where further details on analysis can be found.^1^

This protocol was optimized to capture cells from lymphoid tissue and purify scarce stromal populations to perform transcriptomics analysis. These extremely delicate and rare cells are often underrepresented in published studies. The protocol described here depicts the dissection and digestion of lymphoid tissue to ensure a sufficient yield and viability of stromal populations (Figure 4). Lymph nodes are composed by 99% immune cells and 1% stromal cells. With this protocol single cell suspensions extracted after tissue digestion can be used for in depth analysis of stromal cell populations: FRC, LEC, BEC and DNC (Figure 4). We show that the cells extracted from this digestion can further be analyzed using flow cytometry, in primary cultures or transcriptomics (Figures 4 and 6). Using the gentle automatic magnetic based cell sorting, we achieve a minimal purity of over 99% for the CD45^+^ and CD31^+^ cells (positive separation) and above 80% for the fibroblasts (negative separation – CD45^−^ and CD31^−^) (Figures 5D–5F). After sorting we can achieve viability of over 75% cells for fibroblasts, while for CD45^+^ and CD31^+^ cells viability is above 90% (Figure 5G). Using the 10x Chromium platform to perform scRNA-sequencing of CD45^−^CD31^-^ populations we were able to identify 11 stromal cell populations (Figure 6D).^1^ Our analysis of the CD45^+^CD31^+^ populations identified immune cell populations and endothelial cells (Figure 6E).^1^ The methods described here can advance the field of stromal immunology and enhance research of these rare cells.

## Quantification and statistical analysis

Prism10 Software (GraphPad) was used to create graphs and statistical analysis. Comparisons of two datasets was performed using two-tailed Mann-Whitney tests. For all tests, *p* < 0.05 was considered significant.

## Limitations

The protocol requires digestion of tissues, multiple steps of incubations and cell sorting. Fibroblasts are extremely large and delicate cells and despite all precautions we are unable to achieve a viability above 90% which is typically required for cell-ID barcoding using the 10x Chromium Platform.

## Troubleshooting

### Problem 1

**Figure 7:**
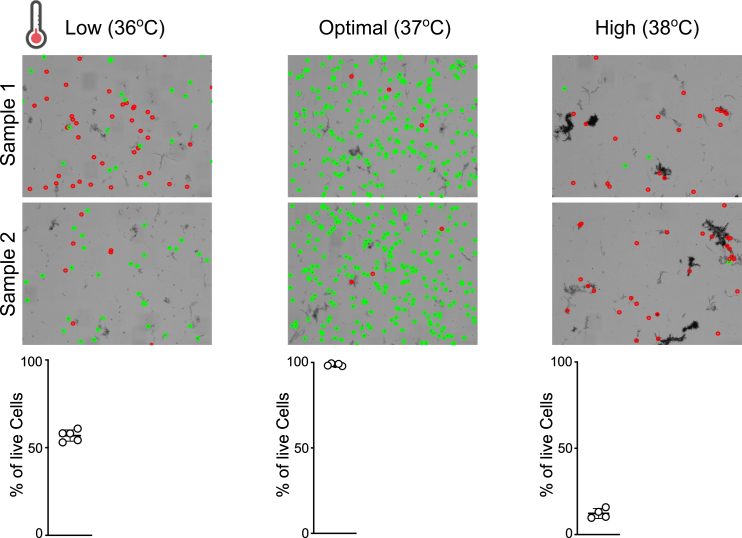
Effect of temperature on viability of lymph node cells Waterbath settings were set at 36°C (low), 37°C (optimal) and 38°C (high) showing viability images and percentages.

Setting the correct water bath temperature for lymph node digestion described in Part 2 is required for optimal enzymatic activity and cell viability of approximately 98–100%. A temperature higher than 37°C will cause increased enzyme activity and reduce cell viability to almost 0%. In contrast temperatures below that will decrease enzymatic activity and prolonged the tissue digestion step leading to a viability of approximately 50%. Both temperature variations will decrease cell viability and yield (Figure 7).

### Potential solution

Even digital water baths can have a variation in temperature, and this can affect the enzymatic digestions of lymph nodes. We recommend using additional thermometers placed at the height of the sample tube (Figure 3C) and constantly monitor the temperature during digestion steps. Temperature can then be adjusted by using the temperature dials. In extreme cases that temperature starts increasing above 37.5°C it can be lowered slightly by adding of small quantities of ice. Maintaining the temperature to 37°C will ensure optimal lymph node digestion and cell recovery (Figure 4).

### Problem 2

**Figure 8:**
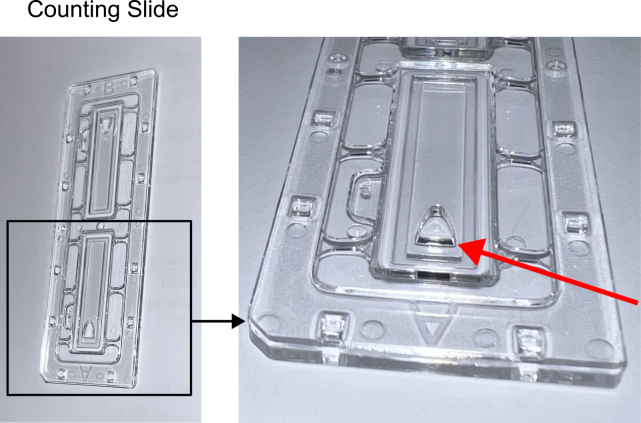
LUNA™ Cell Counting Slides Images show example of the counting slides used in this protocol. The arrow in the zoomed image indicates the location of the loading chamber.

Fibroblasts (negative selection) are larger in size than lymphocytes and very fragile (Step 31). Viability of cells can be greatly affected when loading into counting chambers which are very narrow (Figure 8).

### Potential solution

Load cells onto the counting slides at very slow speed. When loading cells, we also recommend not touching the chamber with pipette tips.

### Problem 3

When dissecting axillary and brachial lymph nodes (Step 3) blood vessels in vicinity can be punctured and blood can surround lymph nodes. This can make identification of lymph nodes during dissection more difficult and can also affect cellular yield after digestion.

### Potential solution

If vessels are punctured blot with tissue until bleeding resides. If necessary, blood can be removed from lymph nodes by washing in RPMI and blotting on Precision Wipes.

### Problem 4

Using cervical dislocation as euthanasia method (Step 1) can increase the presence of blood within these lymph nodes and the surrounding tissue. This can especially compromise the collection of axillary and brachial lymph nodes.

### Potential solution

Use alternative euthanasia methods to such as CO_2_ overdose (Step 1) or intraperitoneal injection of pentobarbital. Cervical dislocation can be used if only inguinal lymph nodes are being collected.>

### Problem 5

Typical digestion times for naïve (non-reactive) lymph nodes is 50 min (Step 9). In experiments involving immunization/infection of mice the lymph nodes are expected to be inflamed (reactive). This results in larger, reactive, lymph nodes^3^^,^^16^ and the tissue may take longer to digest compared to non-reactive experimental groups.

### Potential solution

The digestion of reactive lymph nodes can be prolonged (typically up to 70 min) by repeating Step 9 until all tissue has been digested. When designing experiments involving reactive lymph nodes we recommend preparing an extra 3 mL of Digestion Buffer per sample.

## Resource availability

### Lead contact

Further information and requests for resources and reagents should be directed to and will be fulfilled by the lead contact, Prof. Sophie E. Acton (s.acton@ucl.ac.uk).

### Technical contact

Technical questions on executing this protocol should be directed to and will be answered by the technical contact, Spyridon Makris (spyridon.makris.10@ucl.ac.uk).

### Materials availability

This study did not generate new unique reagents.

### Data and code availability

All data are available from the lead author upon request. This article does not report original code, and scRNA-seq analysis for Figure 6 can be found in Makris et al.^1^

## Acknowledgments

This work has been supported by the 10.13039/100010663European Research Council Starting Grant LNEXPANDS (S.E.A.), 10.13039/501100000289Cancer Research UK Career Development Fellowship CRUK-A19763 (S.E.A.), Rosetrees Trust Foundation PGS22 100023-CD1 (S.E.A.), 10.13039/501100000289Cancer Research UK Senior Fellowship CRUK-RCCSCF-May22\100001 (S.E.A.), and Wellcome Discovery Award 227890/Z/23/Z. We thank the UCL Laboratory for Molecular Cell Biology Light Microscopy Facility for image acquisition and the Central Biological Services for animal maintenance.

## Author contributions

S.M. and S.E.A. conceptualized the protocol. S.M. performed data analysis. S.M., D.S., and A.C.B. performed the experiments for this manuscript. S.M. wrote the manuscript, and S.E.A. assisted in manuscript preparation.

## Declaration of interests

The authors declare no competing interests.

## References

[1] Makris S., Hari-Gupta Y., Cantoral-Rebordinos J.A., Martinez V.G., Horsnell H.L., Benjamin A.C., Cinti I., Jovancheva M., Shewring D., Nguyen N. (2025). Lymph node fibroblast phenotypes and immune crosstalk regulated by podoplanin activity. Cell Rep..

[2] Wilkins A., Makris S., Green M., Hooper S., Mikolajczak A., Giangreco G., Allanki S., Nye E., Connick E., Mitter R. (2025). Rapid expansion of podoplanin-positive fibroblasts following radiation limits the anti-tumour CD8+ T-cell response to radiotherapy. bioRxiv.

[3] Horsnell H.L., Tetley R.J., De Belly H., Makris S., Millward L.J., Benjamin A.C., Heeringa L.A., de Winde C.M., Paluch E.K., Mao Y., Acton S.E. (2022). Lymph node homeostasis and adaptation to immune challenge resolved by fibroblast network mechanics. Nat. Immunol..

[4] Assen F.P., Abe J., Hons M., Hauschild R., Shamipour S., Kaufmann W.A., Costanzo T., Krens G., Brown M., Ludewig B. (2022). Multitier mechanics control stromal adaptations in the swelling lymph node. Nat. Immunol..

[5] Sixt M., Kanazawa N., Selg M., Samson T., Roos G., Reinhardt D.P., Pabst R., Lutz M.B., Sorokin L. (2005). The conduit system transports soluble antigens from the afferent lymph to resident dendritic cells in the T cell area of the lymph node. Immunity.

[6] Acton S.E., Onder L., Novkovic M., Martinez V.G., Ludewig B. (2021). Communication, construction, and fluid control: lymphoid organ fibroblastic reticular cell and conduit networks. Trends Immunol..

[7] Martinez V.G., Pankova V., Krasny L., Singh T., Makris S., White I.J., Benjamin A.C., Dertschnig S., Horsnell H.L., Kriston-Vizi J. (2019). Fibroblastic Reticular Cells Control Conduit Matrix Deposition during Lymph Node Expansion. Cell Rep..

[8] Makris S., de Winde C.M., Horsnell H.L., Cantoral-Rebordinos J.A., Finlay R.E., Acton S.E. (2022). Immune function and dysfunction are determined by lymphoid tissue efficacy. Dis. Model. Mech..

[9] Onder L., Papadopoulou C., Lütge A., Cheng H.-W., Lütge M., Perez-Shibayama C., Gil-Cruz C., Martin A.D., Kurz L., Cadosch N. (2024). Fibroblastic reticular cells generate protective intratumoral T cell environments in lung cancer. Cell.

[10] Fletcher A.L., Malhotra D., Acton S.E., Lukacs-Kornek V., Bellemare-Pelletier A., Curry M., Armant M., Turley S.J. (2011). Reproducible Isolation of Lymph Node Stromal Cells Reveals Site-Dependent Differences in Fibroblastic Reticular Cells. Front. Immunol..

[11] Broggi M.A.S., Schmaler M., Lagarde N., Rossi S.W. (2014). Isolation of Murine Lymph Node Stromal Cells. J. Vis. Exp..

[12] Reeves J.P., Reeves P.A. (2001). Removal of Lymphoid Organs. Curr. Protoc. Im..

[13] Link A., Vogt T.K., Favre S., Britschgi M.R., Acha-Orbea H., Hinz B., Cyster J.G., Luther S.A. (2007). Fibroblastic reticular cells in lymph nodes regulate the homeostasis of naive T cells. Nat. Immunol..

[14] Rodda L.B., Lu E., Bennett M.L., Sokol C.L., Wang X., Luther S.A., Barres B.A., Luster A.D., Ye C.J., Cyster J.G. (2018). Single-Cell RNA Sequencing of Lymph Node Stromal Cells Reveals Niche-Associated Heterogeneity. Immunity.

[15] Schindelin J., Arganda-Carreras I., Frise E., Kaynig V., Longair M., Pietzsch T., Preibisch S., Rueden C., Saalfeld S., Schmid B. (2012). Fiji: an open-source platform for biological-image analysis. Nat. Methods.

[16] Acton S.E., Farrugia A.J., Astarita J.L., Mourão-Sá D., Jenkins R.P., Nye E., Hooper S., van Blijswijk J., Rogers N.C., Snelgrove K.J. (2014). Dendritic cells control fibroblastic reticular network tension and lymph node expansion. Nature.

